# Association of SNPs in *LCP1* and *CTIF* with hearing in 11 year old children: Findings from the Avon Longitudinal Study of Parents and Children (ALSPAC) birth cohort and the G-EAR consortium

**DOI:** 10.1186/s12920-015-0112-2

**Published:** 2015-08-12

**Authors:** Sean Harrison, Sarah J. Lewis, Amanda J. Hall, Dragana Vuckovic, Giorgia Girotto, Richard M. Martin, Josephine C. Adams

**Affiliations:** School of Social and Community Medicine, University of Bristol, Bristol, BS8 2PS UK; Department of Medical, Surgical and Health Sciences, University of Trieste, 34100 Trieste, Italy; School of Biochemistry, University of Bristol, Bristol, BS8 1TD UK

**Keywords:** ALSPAC, Hearing, Otoacoustic emission, Hair cells, Stereocilia, Children

## Abstract

**Background:**

The genetic basis of hearing loss in humans is relatively poorly understood. In recent years, experimental approaches including laboratory studies of early onset hearing loss in inbred mouse strains, or proteomic analyses of hair cells or hair bundles, have suggested new candidate molecules involved in hearing function. However, the relevance of these genes/gene products to hearing function in humans remains unknown. We investigated whether single nucleotide polymorphisms (SNPs) in the human orthologues of genes of interest arising from the above-mentioned studies correlate with hearing function in children.

**Methods:**

577 SNPs from 13 genes were each analysed by linear regression against averaged high (3, 4 and 8 kHz) or low frequency (0.5, 1 and 2 kHz) audiometry data from 4970 children in the Avon Longitudinal Study of Parents and Children (ALSPAC) birth-cohort at age eleven years. Genes found to contain SNPs with low *p*-values were then investigated in 3417 adults in the G-EAR study of hearing.

**Results:**

Genotypic data were available in ALSPAC for a total of 577 SNPs from 13 genes of interest. Two SNPs approached sample-wide significance (pre-specified at *p* = 0.00014): rs12959910 in CBP80/20-dependent translation initiation factor (*CTIF*) for averaged high frequency hearing (*p* = 0.00079, β = 0.61 dB per minor allele); and rs10492452 in L-plastin (*LCP1*) for averaged low frequency hearing (*p* = 0.00056, β = 0.45 dB). For low frequencies, rs9567638 in *LCP1* also enhanced hearing in females (*p* = 0.0011, β = −1.76 dB; males *p* = 0.23, β = 0.61 dB, likelihood-ratio test p = 0.006). SNPs in *LCP1* and *CTIF* were then examined against low and high frequency hearing data for adults in G-EAR. Although the ALSPAC results were not replicated, a SNP in *LCP1*, rs17601960, is in strong LD with rs9967638, and was associated with enhanced low frequency hearing in adult females in G-EAR (*p* = 0.00084).

**Conclusions:**

There was evidence to suggest that multiple SNPs in *CTIF* may contribute a small detrimental effect to hearing, and that a sex-specific locus in *LCP1* is protective of hearing. No individual SNPs reached sample-wide significance in both ALSPAC and G-EAR. This is the first report of a possible association between *LCP1* and hearing function.

**Electronic supplementary material:**

The online version of this article (doi:10.1186/s12920-015-0112-2) contains supplementary material, which is available to authorized users.

## Background

Hearing impairment has a major impact on quality of life. The difficulties associated with progressive hearing loss are particularly apparent with regard to the growing population of older individuals [1, 2]. Nevertheless, even minimal hearing loss in school-age children, which can be detected in around 1 % of children [3], may affect performance at school [4]. It is recognised that progressive hearing loss relates to both genetic and environmental factors, and to the interaction between these factors [5, 6]. Greater knowledge of the genetic factors that contribute to hearing loss could assist early identification of susceptible individuals in the general population.

Over the last 10–15 years, major advances have been made in understanding the molecular basis of mechanotransduction of sound waves in the mammalian inner ear, largely from the study of inherited forms of deafness [7, 8]. In the inner ear, the auditory epithelium contains specialised hair cells with an elaborate morphology in which the apical surfaces are decorated with stereociliary or “hair” bundles. These make contact with the tectorial membrane, which is deflected by incoming sound waves. Each bundle is composed of a group of stereocilia, organised spatially such that they increase in length across the bundle. A protein complex, the tip link complex, is located at the tip of each stereocilium and makes contact with adjacent stereocilia such that the bundle is displaced in a coordinated way upon deflection of the tectorial membrane [7]. Each stereocilium is made rigid by a central bundle of cross-linked actin microfilaments (F-actin) that contains fascin-2 and other actin-binding proteins [7, 9]. Myosins and an intracellular protein complex provide linkage between the tip complex and the F-actin bundle. Movements of the stereocilia activate an as yet unknown mechanotransducer channel and Ca^2+^ ion movements; in inner ear hair cells this initiates intracellular signals and second messengers that activate impulses in adjacent nerve cells [7]. Mutations in multiple components of the tip link complex, for example, cadherin-23, are causal for the deafness/blindness syndrome, Usher syndrome type I [7, 8, 10–12].

A less-utilised route towards the identification of genetic factors associated with hearing in humans is to build on recent studies of the proteome of hair cells and new identifications of hearing-associated genes in inbred strains of laboratory mice [13–15]. The proteomic studies in several species have revealed more comprehensively the complexity of inner ear cells and have identified previously unsuspected protein components of hair bundles [13]. Similarly, studies of early-onset hearing loss in laboratory mice have implicated a number of genes in hearing function for the first time [13, 15]. The relevance of many of these gene products or their encoding genes to human hearing function remains unclear. Furthermore, to our knowledge, genomic association studies of hearing function in humans, to date, have examined only adult populations.

In this study, we selected a suite of genes on the basis of recent published experimental evidence of localisation of their transcripts or protein products in hair cells, or new functional data that associate these genes with hearing function in mice [13–15]. To examine whether single nucleotide polymorphisms (SNPs) in the selected genes are associated with variation in hearing function in children at age 11 years, we analysed the large Avon Longitudinal Study of Parents and Children (ALSPAC) birth cohort [16] for possible associations. The results were further examined and validated with reference to a study of hearing function in European adults from isolated villages that was conducted within the international G-EAR consortium [17].

## Methods

ALSPAC is a population-based prospective cohort study investigating factors that affect the health and development of children and their parents. The study methods are described in detail elsewhere [16, 18]. The study website contains details of all the data that is available through a searchable data dictionary http://www.bris.ac.uk/alspac/researchers/data-access/data-dictionary/. In brief, 14, 541 women who had an expected delivery date between April 1991 and December 1992 were enrolled in the study. 13,988 live born infants survived to at least one year of age. Children eligible for inclusion in our analysis had at least one audiometry test at 11 years of age (*n* = 7082); were of white ethnicity (*n* = 6212); and had DNA genotyping data available (*n* = 4970). The ALSPAC children have been genotyped previously using the Illumina HumanHap550 quad chip (Illumina Inc., San Diego, CA) according to the stated protocol [19]. SNPs with a minor allele frequency of < 1 % or a call rate of < 95 % were removed from the dataset. Ethical approval for the study was obtained from the ALSPAC Ethics and Law Committee and the Local Research Ethics Committees.

Genes of interest were identified on the basis of recent studies of hair cell or hair bundle proteomics and/or studies of early-onset hearing loss in inbred strains of laboratory mice [13–15] and genotyping data for all SNPs listed in these genes in NCBI dbSNP [20] were requested from ALSPAC. Information on SNPs was available for thirteen genes of interest (Table 1, which also indicates the rationale for including each gene in the analysis). *CHD23* and *PCDH15* were included in the set because of their known roles in inherited forms of deafness [11, 12, 21]. SNPs that had been genotyped within ALSPAC were made available in anonymised form (ALSPAC service level agreement B1480). Genotypic dosage for all SNPs of interest, which represents the expected number of the rare (SNP) allele in the range from 0 to 2 (where 0 is the most common allele and 2 represents homozygous for the rare allele), was used in the association analysis. Genotypes were checked for deviation from the Hardy-Weinberg equilibrium using the hwsnp function implemented in Stata (StataCorp LP, 2012, College Station, TX). Any SNP with evidence of violations of Hardy-Weinberg equilibrium (p < 8.65×10^−5^), >5 % missing, or incorrect imputations, (as defined by a genotypic dosage more than 0.05 away from 0, 1 or 2), was discarded.

**Table 1:** Summary of the genes analysed in this study

Gene acronym	Full gene name	Known association of mutations with human audiological condition	Reference
*CDH23*	Cadherin-23	Non-syndromic deafness, early-onset deafness	[11, 12]
*ESPNL*	Espin-like	None reported; protein present in hair bundles (rat and chick)	[13]
*FSCN2*	Fascin-2	None reported; *Fscn2* mutation associated with early onset ≤ 16 kHz hearing loss in C57BL/6 J mice *(ahl8);* protein present in hair bundles (rat, chick, zebrafish)	[8, 13, 32]
*HCN2*	hyperpolarization activated cyclic nucleotide-gated potassium channel 2	None reported; expressed in cochlear hair cells (mouse, rat)	[46, 47]
*LCP1*	Plastin-2/L-plastin	None reported; protein present in hair bundles (chick)	[13]
*PDCH15*	protocadherin-15	Non-syndromic deafness, Usher Syndrome type 1 F	[6–8, 21]
*XIRP2*	xin actin-binding repeat containing 2	None reported; protein present in hair bundles (rat and chick)	[13]
*ACAA2*	acetyl-CoA acyltransferase 2	None reported; *ACAA2, c18orf32, CTIF*, *DYM, MYO5B* and *SMAD7* are human orthologues of six genes in the *ahl9* QTL on mouse chromosome 18 that is associated with low frequency hearing loss in BXD recombinant inbred mice by 2–3 months of age.	[15]
*c18orf32*	chromosome 18 open reading frame 32		
*CTIF*	CBP80/20-dependent translation initiation factor		
*DYM*	dymeclin		
*MYO5B*	myosin VB		
*SMAD7*	SMAD family member 7		

### Population stratification

The top 10 principal components (PCs) that reflect the genetic structure of the population were estimated according to Price et al. [22] from genome-wide SNPs genotyped, imputed and cleaned in ALSPAC children, as described above. All 10 PCs were included as covariates in the regression models to account for confounding by population stratification.

### Measures of hearing function

Hearing function of the cohort was examined using air conduction pure tone audiometry and an objective measure of cochlear function, otoacoustic emissions (OAE), at age 11 years. All tests were conducted by audiologists or trained physiology staff and measurements were carried out as described in Hall et al., 2012 [23]. Hearing thresholds were measured in both ears at 0.5 – 8 kHz according to the British Society of Audiology recommended procedure for audiometry [24]. For our analysis, the lowest threshold for each frequency, i.e., the ear with the best hearing, was used to create 2 new pure tone averages (PTA) by taking the PTA of the high/medium (3, 4 and 8 kHz) or the low/medium (0.5, 1 and 2 kHz) frequency hearing thresholds. This is standard practice in epidemiological studies of hearing [25].

Transient evoked OAE were measured in both ears and the OAE response level was measured from the unfiltered recordings and at 1, 2, 3 and 4 kHz as described in detail in Hall et al., 2012 [23]. In our analysis, to preserve statistical power, the unfiltered (broadband) recordings were used. Large OAE amplitudes are associated with better hearing function. The hearing levels of the ALSPAC subjects are listed in Table 2.

**Table 2:** Summary of hearing threshold levels and otoacoustic emission amplitudes in ALSPAC children

Measure	Frequency (kHz)	Gender	Number	Mean (SD)	Range (dB)	*N* > 40 dB (%)
Audiometry	0.5	All	4931	2.93 (6.0)	−10 to 80	12 (0.24)
	1	All	4970	1.40 (6.0)	−10 to 95	10 (0.20)
	2	All	4969	1.38 (6.2)	−10 to 110	10 (0.20)
	3	All	4936	1.22 (6.3)	−10 to 110	10 (0.20)
	4	All	4964	1.07 (6.6)	−10 to 100	10 (0.20)
	8	All	4950	4.57 (8.0)	−10 to 65	17 (0.34)
	High frequency	All	4923	2.27 (5.6)	−10 to 70	7 (0.14)
	Low frequency	All	4931	1.90 (5.2)	−10 to 95	10 (0.20)
OAE	Broadband	All	3711	13.78 (5.5)	−9 to 31	-
	1	All	3711	8.36 (7.0)	−13 to 26	-
	2	All	3711	6.90 (6.6)	−17 to 28	-
	3	All	3711	4.21 (7.2)	−17 to 28	-
	4	All	3711	0.35 (7.3)	−21 to 24	-
Audiometry	0.5	Males	2421	2.96 (5.8)	−10 to 60	5 (0.21)
	1	Males	2445	1.55 (5.8)	−10 to 65	4 (0.16)
	2	Males	2444	1.49 (6.0)	−10 to 70	3 (0.12)
	3	Males	2426	1.57 (6.0)	−10 to 75	4 (0.16)
	4	Males	2441	1.20 (6.7)	−10 to 75	5 (0.20)
	8	Males	2436	5.01 (8.2)	−10 to 65	9 (0.37)
	High frequency	Males	2421	2.59 (5.7)	−10 to 70	3 (0.12)
	Low frequency	Males	2421	1.98 (5.0)	−8 to 62	3 (0.12)
OAE	Broadband	Males	1848	12.90 (5.4)	−9 to 31	-
	1	Males	1848	7.56 (6.9)	−13 to 26	-
	2	Males	1848	5.87 (6.5)	−17 to 25	-
	3	Males	1848	3.00 (7.0)	−17 to 28	-
	4	Males	1848	−0.53 (7.1)	−21 to 24	-
Audiometry	0.5	Females	2510	2.91 (6.2)	−10 to 80	7 (0.28)
	1	Females	2525	1.25 (6.2)	−10 to 95	6 (0.24)
	2	Females	2525	1.27 (6.3)	−10 to 110	7 (0.28)
	3	Females	2510	0.89 (6.6)	−10 to 110	6 (0.24)
	4	Females	2523	0.95 (6.5)	−10 to 100	5 (0.20)
	8	Females	2514	4.14 (7.8)	−10 to 55	8 (0.32)
	High frequency	Females	2502	1.96 (5.5)	−10 to 57	4 (0.16)
	Low frequency	Females	2510	1.82 (5.5)	−10 to 95	7 (0.28)
OAE	Broadband	Females	1863	14.65 (5.5)	−5 to 29	-
	1	Females	1863	9.15 (6.9)	−13 to 26	-
	2	Females	1863	7.92 (6.5)	−15 to 28	-
	3	Females	1863	5.42 (7.3)	−16 to 23	-
	4	Females	1863	1.22 (7.4)	−21 to 22	-

### Statistical methods

Haploview was used to calculate the number of LD blocks in the SNPs studied, using the solid spine of LD option [26]. A Bonferroni correction was applied by using the number of LD blocks across all the SNPs studied as an estimate of the number of independent tests in the sample, because linkage disequilibrium in association studies results in non-independent tests and an over-correction for Type 1 errors [27]. This method of correction has been criticised as insufficiently conservative for genome-wide association studies, but our study uses a limited number of SNPs from a relatively small set of genes [28].

Linear regression was used to determine if any of the SNPs correlated with an effect on the high- or low-frequency PTAs or the broadband OAE results, using the 10 principal components as co-variables. The analyses were also stratified by gender, because evidence from our validation study (see below) suggested that the observed effects were gender specific. The *a priori* threshold for significance to which the *p*-values were compared was calculated as 0.05/([the number of frequencies tested]*[the number of independent tests]); however, it is noted that this may still be conservative because the high and low frequency PTAs might be associated, making them non-independent tests. All analyses were carried out in Stata version 13 (StataCorp LP, 2012, College Station, TX). In the reporting of the effect sizes of the minor (SNP) alleles, a positive value represents reduced hearing and a negative value represents enhanced hearing.

### External validation and combination of data

The most interesting results from our analysis of 11-year old children in ALSPAC were compared with an external genome-wide association study (GWAS) G-EAR, on the basis of the *p*-values and directions of effects. The full G-EAR study is described elsewhere [17], briefly, 3417 subjects within G-EAR aged 18 years or older were recruited from isolated villages in Italy and Croatia and underwent pure-tone audiometry, tympanometry, and acoustic reflex testing in both ears after any acoustically obstructing ear-wax had been removed. Analysis of hearing function was undertaken by calculating the PTA of air conduction using the best ear at the lower (0.25, 0.5, and 1 kHz), medium (0.5, 1, and 2 kHz), and high frequencies (4, 8 kHz). Each trait was regressed against age, sex and genomic kinship prior to analysis. For our study, the audiometry results in G-EAR were rank transformed because the data were skewed, unlike the data from ALSPAC which were approximately normally distributed.

The rank transformation of the G-EAR data limited the possible merging of results and comparison of effect sizes: rank transformations rank the data smallest to largest and force each data point’s rank value to a normal distribution with minimum value 0 and maximum value 1. Thus, the effect sizes in the G-EAR study related to an artificially created normal curve, but they were consistent with the ALSPAC study: i.e., positive effects imply worse hearing function.

### Genomic loci of SNPs

The position of SNPs of interest within *CTIF* and *LCP1* was examined with reference to dbSNP at NCBI and by BLASTN searches of the NCBI human genome GRCh38 primary assembly [29]. SNP positions were identified on reference assembly annotation release 105 using the table of reference sequence transcripts in the Map view function. Diagrams of gene structure for *LCP1* and *CTIF* were prepared from exports from Ensembl 2014 [30], (*CTIF* from entry ENSG00000134030 and *LCP1* from entry ENSG00000136167), and are presented in fancyGENE 1.4 [31].

## Results

### Identification of SNPs in *CTIF* and *LCP1* that correlate with altered hearing in ALSPAC children

For the 7082 children for whom hearing data had been obtained at 11 years of age, 4970 (70.2 %) were of white ethnicity and had full genotypic data. 49 % (n = 2445) of the children were male. The data on hearing function for the 4970 children are summarised in Table 2. Genotypic data were available for 602 SNPs from the 13 genes of interest: 24 SNPs had >5 % incorrect imputations (defined as being >0.05 away from a whole number, where 0 was homozygous for the major allele, 1 was heterozygous and 2 was homozygous for the minor allele) and were removed and 1 SNP was out of HW equilibrium (no minor allele homozygotes), leaving a total of 577 SNPs from 13 genes for our analysis (Table 3).

**Table 3:** Summary of the study SNPs from ALSPAC

Gene	Chromosome	Number of SNPs	Number of LD Blocks
*ACAA2*	18	4	1
*C18ORF32*	18	1	1
*CDH23*	10	7	2
*CTIF*	18	111	31
*DYM*	18	51	5
*ESPNL*	2	5	2
*FSCN2*	17	1	1
*HCN2*	19	3	2
*LCP1*	13	18	3
*MYO5B*	18	114	15
*PCDH15*	10	210	32
*SMAD7*	18	13	6
*XIRP2*	2	39	17
Total		577	118

Haploview’s solid spine of LD computed 118 groups of LD, which gave a sample-wide SNP threshold *p*-value of 0.00014 [0.05/(3*118) = 0.00014]. The effect size (β) of the regression represents the decibel (dB) change in the hearing threshold for the averaged frequencies for each copy of the minor allele; an increase indicates a detrimental effect on hearing function.

Two SNPs in the analysis achieved near-significance at the sample-wide level: rs12959910 in *CTIF* for the high frequency PTA (*p* = 0.00079, β = 0.61 dB, 95 % CI: 0.26-0.97) and rs10492452 in *LCP1* for the low frequency PTA (*p* = 0.00056, β = 0.45 dB, 95 % CI: 0.20-0.71) (Table 4 and Additional file 1).

**Table 4:** Effect sizes of SNPs with the lowest *p*-values from the analysis of ALSPAC children

10 most significant SNPs associated with high frequency hearing overall (max *n* = 4970)								
SNP	Chromosome	Base Position	Gene	Effect Size (dB)	*p*-value	LD Block	Minor allele	MAF
rs12959910	18	46337861	*CTIF*	0.61	0.00079	19	G	0.11
rs8085434	18	46200968	*CTIF*	0.84	0.0024	8	C	0.05
rs8091955	18	46358907	*CTIF*	0.34	0.0036	22	G	0.45
rs1994559	18	46332358	*CTIF*	0.43	0.0043	19	A	0.17
rs1319946	18	46359024	*CTIF*	0.31	0.0067	22	C	0.49
rs1877192	2	167863420	*XIRP2*	0.43	0.014	3	C	0.12
rs10825335	10	56234349	*PCDH15*	0.41	0.022	18	A	0.11
rs1317625	18	46380182	*CTIF*	0.3	0.026	25	A	0.22
rs7233521	18	46219733	*CTIF*	0.43	0.031	11	A	0.09
rs1316826	18	46348156	*CTIF*	0.26	0.032	18	C	0.31
10 most significant SNPs associated with high frequency hearing in males (max *n* = 2445)								
SNP	Chromosome	Base Position	Gene	Effect Size (dB)	*p*-value	LD Block	Minor allele	MAF
rs2337069	18	46120940	*CTIF*	0.58	0.0041	3	T	0.22
rs16951446	18	47599093	*MYO5B*	−1.06	0.0047	8	G	0.05
rs16951488	18	47610821	*MYO5B*	−1.06	0.0047	8	G	0.05
rs8085434	18	46200968	*CTIF*	1.04	0.0093	8	C	0.05
rs10823837	10	73498910	*CDH23*	0.44	0.0094	1	C	0.43
rs1877192	2	167863420	*XIRP2*	0.65	0.011	3	C	0.12
rs11662494	18	46241017	*CTIF*	−0.57	0.012	13	T	0.15
rs10825335	10	56234349	*PCDH15*	0.67	0.012	18	A	0.11
rs869000	2	167862974	*XIRP2*	0.74	0.019	3	C	0.07
rs4939612	18	47541612	*MYO5B*	0.52	0.022	7	T	0.16
10 most significant SNPs associated with high frequency hearing in females (max *n* = 2525)								
SNP	Chromosome	Base Position	Gene	Effect Size (dB)	*p*-value	LD Block	Minor allele	MAF
rs4592338	10	56098424	*PCDH15*	1.21	0.0027	16	A	0.04
rs978674	10	56119975	*PCDH15*	1.17	0.0032	16	A	0.04
rs7321994	13	46742539	*LCP1*	−0.53	0.0055	2	A	0.20
rs1787534	18	47445407	*MYO5B*	0.75	0.0055	1	T	0.09
rs873816	18	46101074	*CTIF*	−0.59	0.0079	2	C	0.15
rs9567638	13	46754734	*LCP1*	−1.43	0.0084	3	C	0.02
rs8087713	18	46677185	*DYM*	0.54	0.011	2	G	0.17
rs10775489	18	46667299	*DYM*	0.54	0.011	2	A	0.17
rs12959910	18	46337861	*CTIF*	0.64	0.012	19	G	0.10
rs2296119	13	46729259	*LCP1*	−0.49	0.015	2	T	0.17
10 most significant SNPs associated with low frequency hearing overall (max *n* = 4970)								
SNP	Chromosome	Base Position	Gene	Effect Size (dB)	*p*-value	LD Block	Minor allele	MAF
rs10492452	13	46721562	*LCP1*	0.45	0.00056	1	A	0.20
rs16951446	18	47599093	*MYO5B*	−0.73	0.0017	1	G	0.05
rs16951488	18	47610821	*MYO5B*	−0.73	0.0017	1	G	0.05
rs16913796	10	55659824	*PCDH15*	−0.59	0.003	3	T	0.08
rs6561296	13	46702401	*LCP1*	0.38	0.0033	1	C	0.21
rs9316187	13	46713702	*LCP1*	0.33	0.0079	1	A	0.24
rs6432974	2	167982976	*XIRP2*	0.39	0.0097	5	T	0.14
rs1937389	10	56319852	*PCDH15*	0.37	0.01	24	G	0.16
rs11003924	10	55662156	*PCDH15*	−0.44	0.01	4	T	0.11
rs11003925	10	55662226	*PCDH15*	−0.44	0.01	4	T	0.11
10 most significant SNPs associated with low frequency hearing in males (max *n* = 2445)								
SNP	Chromosome	Base Position	Gene	Effect Size (dB)	*p*-value	LD Block	Minor allele	MAF
rs1877192	2	167863420	*XIRP2*	0.69	0.0017	3	C	0.12
rs12571150	10	56546055	*PCDH15*	−0.51	0.0022	29	T	0.25
rs16951446	18	47599093	*MYO5B*	−0.89	0.0062	1	G	0.05
rs16951488	18	47610821	*MYO5B*	−0.89	0.0062	1	G	0.05
rs1912985	10	56538759	*PCDH15*	0.53	0.0068	28	T	0.16
rs10492452	13	46721562	*LCP1*	0.48	0.008	1	A	0.20
rs1317355	18	46379626	*CTIF*	0.45	0.0086	24	T	0.23
rs877885	18	46241163	*CTIF*	0.42	0.0097	13	A	0.26
rs11662494	18	46241017	*CTIF*	−0.51	0.0098	13	T	0.15
rs937023	18	46383785	*CTIF*	0.41	0.011	25	C	0.28
10 most significant SNPs associated with low frequency hearing in females (max *n* = 2525)								
SNP	Chromosome	Base Position	Gene	Effect Size (dB)	*p*-value	LD Block	Minor allele	MAF
rs7233521	18	46219733	*CTIF*	0.98	0.00047	11	A	0.09
rs9567638	13	46754734	*LCP1*	−1.76	0.0011	3	C	0.02
rs7321994	13	46742539	*LCP1*	−0.55	0.0039	2	A	0.20
rs11003876	10	55594049	*PCDH15*	0.45	0.004	1	C	0.43
rs11003889	10	55623229	*PCDH15*	0.49	0.0057	1	C	0.27
rs1937389	10	56319852	*PCDH15*	0.58	0.006	24	G	0.16
rs2296119	13	46729259	*LCP1*	−0.55	0.0062	2	T	0.17
rs4478893	10	55635024	*PCDH15*	0.43	0.0087	2	G	0.34
rs1900425	10	55638740	*PCDH15*	0.42	0.0093	3	A	0.34
rs8087713	18	46677185	*DYM*	0.54	0.01	2	G	0.17

LD block refers to the numbered LD blocks that were calculated using a solid spine of LD in Haploview. Effect size is reported per copy of minor allele in the SNP, and represents the number of decibels (dB) higher or lower for the hearing threshold of children with the minor allele. A positive effect size represents a decreased hearing threshold and a negative effect size represents enhanced hearing. Some SNPs are below the maximum n because not all SNPs in our study were generated for each child. MAF = minor allele frequency

### *CTIF* SNPs and analysis of high frequency hearing function in ALSPAC children

In the analysis of high-frequency hearing function, eight out of the top ten smallest *p*-values were from SNPs in *CTIF* (Table 4). This finding, combined with the very low *p*-value of rs12959910, prompted us to seek validation for the results with *CTIF* for high frequencies in the G-EAR cohort. No other gene in either the non-stratified or the subgroup analyses warranted further investigation from the analysis of high-frequency hearing function (Additional file 1).

### Examination of *CTIF* SNPs and high frequency hearing function in the G-EAR adult cohort

To our knowledge, a GWAS of hearing function in children has not been conducted. Therefore, we attempted validation of our findings from ALSPAC within the G-EAR cohort of adults over 18 years of age in isolated European populations, which had been designed to assess the hearing function and thresholds of isolated European populations within the international G-EAR consortium [17]. The 10 lowest *p*-values from the ALSPAC results for SNPs in *CTIF* are reported in Table 5 with the corresponding *p*-value from G-EAR. No SNP was found to have a clear effect in either the non-stratified or the sub-group analyses. The low *p*-value of rs12959910 was not replicated in the G-EAR cohort (*p* = 0.11).

**Table 5:** Effect sizes of SNPs in *CTIF* or *LCP1* for averaged high or low frequency hearing compared between ALSPAC and G-EAR

*CTIF:* 10 most significant SNPs in high frequency tests overall								
SNP	Minor Allele	Major allele	ALSPAC MAF	ALSPAC Effect Size (dB)	ALSPAC *p*-value	G-EAR Effect Size (rank normal)	G-EAR *p*-value	LD Block
rs12959910	G	A	0.11	0.61	0.0008	0.096	0.11	19
rs8085434	C	T	0.05	0.84	0.002	0.081	0.27	8
rs8091955	G	A	0.45	0.34	0.004	0.045	0.22	22
rs1994559	A	G	0.17	0.43	0.004	0.059	0.23	19
rs1319946	C	T	0.49	0.31	0.007	0.040	0.24	22
rs1317625	A	G	0.22	0.30	0.026	−0.021	0.59	25
rs7233521	A	G	0.09	0.43	0.031	0.069	0.35	11
rs1316826	C	T	0.31	0.26	0.032	0.059	0.17	21
rs4583322	A	G	0.35	0.25	0.032	0.008	0.82	19
rs937021	G	A	0.44	0.23	0.039	−0.043	0.21	25
*CTIF:* 10 most significant SNPs in high frequency tests in males								
SNP	Minor Allele	Major allele	ALSPAC MAF	ALSPAC Effect Size (dB)	ALSPAC *p*-value	G-EAR Effect Size (rank normal)	G-EAR *p*-value	LD Block
rs2337069	T	C	0.22	0.58	0.004	0.010	0.26	3
rs8085434	C	T	0.05	1.04	0.009	0.002	0.89	8
rs11662494	T	C	0.15	−0.57	0.012	0.001	0.91	13
rs937021	G	A	0.45	0.37	0.026	0.003	0.74	25
rs12959910	G	A	0.11	0.57	0.030	0.021	0.10	19
rs7227797	G	A	0.31	0.38	0.033	0.014	0.07	3
rs1994559	A	G	0.18	0.44	0.040	0.002	0.82	19
rs11082695	A	G	0.17	−0.43	0.048	0.001	0.95	13
rs8091955	G	A	0.46	0.32	0.058	0.014	0.08	22
rs1319946	C	T	0.50	0.32	0.060	0.009	0.20	22
*CTIF:* 10 most significant SNPs in high frequency tests in females								
SNP	Minor Allele	Major allele	ALSPAC MAF	ALSPAC Effect Size (dB)	ALSPAC *p*-value	G-EAR Effect Size (rank normal)	G-EAR *p*-value	LD Block
rs873816	C	T	0.15	−0.59	0.008	−0.003	0.69	2
rs12959910	G	A	0.10	0.64	0.012	−0.007	0.55	19
rs8091955	G	A	0.45	0.33	0.036	0.001	0.86	22
rs4939781	G	A	0.21	−0.40	0.040	−0.009	0.20	2
rs11082698	G	A	0.46	0.32	0.043	−0.008	0.18	14
rs1317625	A	G	0.21	0.38	0.047	−0.001	0.83	25
rs4939804	A	G	0.23	0.36	0.053	−0.004	0.60	14
rs11662760	A	G	0.09	−0.53	0.056	−0.006	0.58	1
rs7233521	A	G	0.09	0.54	0.058	−0.028	0.04	11
rs1319946	C	T	0.49	0.30	0.062	−0.001	0.90	22
*LCP1:* 10 most significant SNPs in low frequency tests overall								
SNP	Minor Allele	Major allele	ALSPAC MAF	ALSPAC Effect Size (dB)	ALSPAC *p*-value	G-EAR Effect Size (rank normal)	G-EAR *p*-value	LD Block
rs10492452	A	C	0.20	0.45	0.0006	−0.009	0.86	1
rs6561296	C	T	0.21	0.38	0.003	−0.013	0.79	1
rs9316187	A	G	0.24	0.33	0.008	0.007	0.87	1
rs1409437	G	A	0.44	−0.23	0.029	−0.066	0.08	1
rs2146880	A	C	0.45	0.23	0.030	0.025	0.52	1
rs1886040	C	T	0.49	0.22	0.039	0.028	0.47	1
rs2093707	A	C	0.49	0.21	0.046	0.034	0.37	1
rs7321994	A	G	0.20	−0.24	0.067	−0.047	0.25	2
rs2209093	C	T	0.17	−0.25	0.074	−0.071	0.09	2
rs2296119	T	C	0.17	−0.25	0.076	−0.032	0.46	2
*LCP1:* 10 most significant SNPs in low frequency tests in males								
SNP	Minor Allele	Major allele	ALSPAC MAF	ALSPAC Effect Size (dB)	ALSPAC *p*-value	G-EAR Effect Size (rank normal)	G-EAR *p*-value	LD Block
rs10492452	A	C	0.20	0.48	0.008	−0.004	0.67	1
rs6561296	C	T	0.21	0.40	0.026	−0.006	0.48	1
rs9316187	A	G	0.24	0.37	0.031	−0.004	0.63	1
rs1409437	G	A	0.44	−0.30	0.039	0.003	0.60	1
rs2146880	A	C	0.45	0.26	0.072	−0.001	0.89	1
rs1886040	C	T	0.49	0.26	0.075	−0.003	0.56	1
rs2093707	A	C	0.49	0.25	0.081	−0.003	0.66	1
rs17601960	C	T	0.08	−0.36	0.168	0.043	0.01	3
rs9567638	C	T	0.02	0.59	0.246	0.024	0.48	3
rs10492449	G	T	0.33	0.14	0.373	−0.006	0.33	3
*LCP1:* 10 most significant SNPs in low frequency tests in females								
SNP	Minor Allele	Major allele	ALSPAC MAF	ALSPAC Effect Size (dB)	ALSPAC *p*-value	G-EAR Effect Size (rank normal)	G-EAR *p*-value	LD Block
rs9567638	C	T	0.02	−1.76	0.0011	0.021	0.53	3
rs7321994	A	G	0.20	−0.55	0.004	0.006	0.19	2
rs2296119	T	C	0.17	−0.55	0.006	0.005	0.35	2
rs2209093	C	T	0.17	−0.50	0.012	0.005	0.27	2
rs10492452	A	C	0.21	0.43	0.026	0.003	0.64	1
rs2209092	G	A	0.22	−0.37	0.042	0.006	0.17	2
rs6561296	C	T	0.22	0.36	0.056	0.004	0.55	1
rs9316187	A	G	0.24	0.29	0.110	0.007	0.25	1
rs2146880	A	C	0.45	0.20	0.202	0.000	0.97	1
rs1886040	C	T	0.49	0.18	0.247	0.003	0.55	1

LD block refers to the numbered LD blocks that were calculated using a solid spine of LD in Haploview. The effect allele in G-EAR was the same as in ALSPAC, but the effect sizes in G-EAR were calculated using a rank normal transformation (see Methods) and so cannot be compared directly with the effect sizes from ALSPAC. MAF = minor allele frequency in ALSPAC

### *CTIF* SNPs and analysis of otoacoustic emissions data from ALSPAC children and in the G-EAR cohort

From the 13 genes examined, only one SNP, rs7233521 in *CTIF*, showed a relationship with broadband OAE in the non-stratified and sub-group analyses of the ALSPAC cohort. The effect was specific to females and correlated with smaller amplitude OAE, i.e., poorer hearing function (females, *p* = 0.00000037, β = −1.64 dB, 95 % CI: −2.27 to −1.01; males, *p* = 0.46, β = 0.22 dB, 95 % CI: −0.36-0.80) (see Additional file 1 for OAE results). Notably, this SNP had the lowest *p*-value in females for low frequency hearing function (Table 4). No other SNP in *CTIF* had an effect on low frequency hearing in the ALSPAC cohort. However, in G-EAR, a possible sex-specific effect of rs7233521 on high frequency hearing was noted (*p* = 0.04 in females, *p* = 0.32 in males) (Additional file 1).

### *LCP1* SNPs and analysis of low frequency hearing function in ALSPAC children

In the non-stratified analysis of low frequency hearing for the ALSPAC children, 3 of the 10 lowest *p*-values were from SNPs in the *LCP1* gene, including the SNP with the lowest *p*-value in our analysis, rs10492452 (*p* = 0.00056, β = 0.45 dB, 95 % CI: 0.20-0.71) (Table 4). Furthermore, *LCP1* seemed to have some gender specificity, as the *LCP1* SNP with the largest effect size, rs9567638, had a negative, (i.e., enhanced hearing), effect in girls (*p* = 0.0011, β = −1.76 dB, 95 % CI: −2.81 to −0.71) (Table 4) but a positive (i.e., reduced hearing) or null effect in boys (*p* = 0.23, β = 0.61 dB, 95 % CI: −0.41-1.6, likelihood ratio test *p* = 0.006). There were no differences in the proportion of minor (SNP) alleles between the genders (Tables 4 and 5). No other gene warranted further investigation in either the overall or the subgroup analyses of the low frequency hearing data in ALSPAC children.

### Examination of *LCP1* SNPs and low frequency hearing function in the G-EAR adult cohort

Given that only 3 % of the SNPs (18/577) under investigation are in *LCP1*, the above possible association prompted us to attempt to validate the *LCP1* results for low frequency hearing against the G-EAR cohort. A notable result was obtained from the female subjects in G-EAR: rs17601960 in *LCP1* had a large, sex-specific effect for averaged low frequencies of hearing. These included 0.125 kHz, a frequency that was not studied in ALSPAC (*p* = 0.0008432, see Additional file 1). This SNP is in the same LD group as rs9567638, which correlated with a large effect on low hearing frequencies in girls in ALSPAC (Table 4). Both SNPs have low minor allele frequencies (rs9567638 = 0.02, rs17601960 = 0.036), and reduce (i.e., improve) the low frequency hearing threshold. No other results were replicated in G-EAR (Table 5).

### Genomic loci of identified SNPs of interest in *CTIF* and *LCP1*

**Fig. 1:**
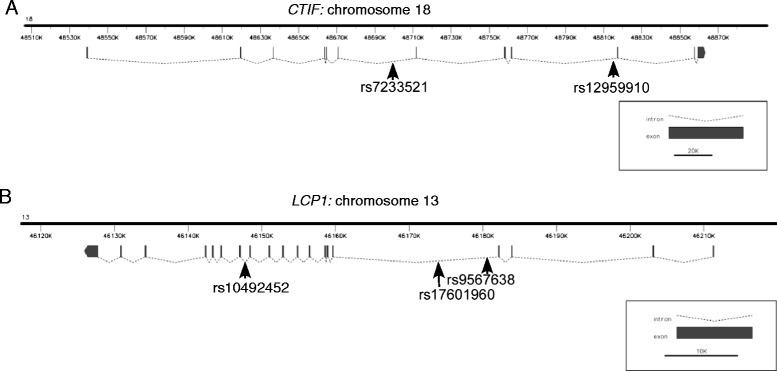
Genomic loci of SNPs in *CTIF* and *LCP1* found to correlate with altered hearing function in children and/or adult women. **a**, diagram of *CTIF* with the positions of implicated SNPs marked. **b**, diagram of *LCP1* with the positions of implicated SNPs marked

The genomic loci of the SNPs of most interest from *CTIF* and *LCP1* were identified in dbSNP and examined with regard to the human genome reference assembly. All five SNPs of interest: rs12959910 and rs7233521 in *CTIF* and rs10492452, rs17601960 and rs9567638 in *LCP1*, are intronic variants. The two SNPs in *CTIF* have distinct locations within the gene (Fig. 1a). Interestingly, rs17601960 and rs9567638 in *LCP1*, which are in the same LD group, are located in the same large intron. SNP rs10492452 has a distinct location in intron 12–13 (Fig. 1b).

## Discussion

This study demonstrates for the first time that SNPs in *CTIF* and *LCP1* correlate with effects on hearing function in children at age 11. No individual SNP achieved our *a priori* overall sample-wide significance threshold, despite the size of the sample studied, although single SNPs from *LCP1* and *CTIF* came close to this threshold. External validation against an adult population did not confirm a general involvement of *CTIF* or *LCP1* in hearing function in adults. However, there was evidence of many SNPs in *CTIF* being detrimental to high frequency hearing in children, and evidence of a specific LD block in *LCP1* being correlated with enhanced low frequency hearing in girls and adult women. No effect was observed on otoacoustic emissions, except for SNP rs7233521 in *CTIF* in females; this SNP had the lowest *p*-value for low frequency hearing results in ALSPAC. This SNP was not validated in the analysis of data from G-EAR. Nevertheless, given the extremely low *p*-value obtained in the ALSPAC cohort, this SNP may be a worthwhile target for future research.

In mice, *Ctif* is within the interval of *ahl9,* a quantitative trait locus correlated with early-onset, low frequency (4 kHz) hearing loss in BXD recombinant inbred mice [15]: mice hear between 1 to 70 kHz, whereas humans hear between 20 Hz to 20 kHz. So 4 kHz is “low frequency” for mice but not for humans. *Ctif* was demonstrated to be expressed in the mouse cochlea with an expression level that is influenced by the parental allele, but the exact sites of expression have yet to be established [15]. The CTIF protein is peri-nuclear and is expressed in many cell types. It is a component of the CBP80/20 translation initiation complex that has a role in nonsense-mediated mRNA decay [33]. Thus, alterations in *CTIF* levels or functionality could potentially affect many target mRNAs. Further research will be needed to establish the expression patterns and functional significance of *CTIF* in the inner ear.

The protein product of *LCP1*, designated plastin-2 or L-plastin, is a member of the plastin family of actin-binding proteins. The mammalian family also includes plastin-1, also known as I-plastin or fimbrin, which is highly expressed in intestine and kidney, and plastin-3 or T-plastin, which is expressed in most solid tissues [34, 35]. Plastin-2 has been characterised principally as a protein present in cells of haematopoietic lineages. In leukocytes, plastin-2 interacts with LFA-1 integrin and is important for leukocyte polarisation, migration and innate and adaptive immune responses. *Lcp1−/−* mice are viable but show defects in B cell development and immune responses [36, 37]. Up-regulation of plastin-2 occurs in various human cancers and a coding SNP in *LCP1* has been correlated with gender- and tumour-stage specific prognostic significance in colorectal cancer recurrence [38, 39]. There are multiple reports that plastin-1 and plastin-3 are present in hair cells; specifically, in the F-actin bundles of stereocilia. Whereas plastin-1 is present in mature stereocilia, plastin-3 has been detected transiently in rat cochlea during hair cell differentiation [40–42]. Subsequent proteomics of hair bundles purified from chicken utricles demonstrated that plastin-1 and fascin-2 are the most abundant cross-linking proteins in these bundles; in addition, both plastin-2 and plastin-3 are present in low abundance [13, 32]. To our knowledge, these are the only data on plastin-2 in hair cells. Recently, plastin-1 (*Pls1*) gene knockout mice were found to have moderate, progressive hearing loss across all frequencies that correlated with morphological abnormalities of stereocilia in mature hair cells [43]. Investigations of *Lcp1* gene knockout mice have focused on immunological functions.

In our study, the SNPs in *LCP1* appeared to be sex-specific, were protective and had low minor allele frequencies, which could possibly represent a relatively new set of mutations. Because these are intronic SNPs, it is possible that their effects relate to mRNA stability. It would be helpful to conduct audiometry testing on *Lcp1* knockout mice to determine if there is a causal relationship between *Lcp1* and hearing function.

*CTIF* had an influence on high frequency but not low frequency hearing, whereas *LCP1* affected only low frequency hearing; these differential frequency effects will require further investigation. It is interesting that the associations were observed with audiometry but not with otoacoustic emissions. Otoacoustic emissions are sensitive to the cochlear amplification function of the outer hair cells [44]. In this study, the lack of an association with OAEs implies that there is no measurable effect of the genes studied on outer hair cell cochlear amplification processes, with the possible exception of rs7233521 in *CTIF* in females.

*CDH23* or *PCDH15* were included in our study set of genes due to their known causal roles in inherited forms of deafness [1, 3, 24]. Whereas SNPs in *PCDH15* were amongst those with the lowest *p* values for effects on high or low frequency hearing in ALSPAC (Table 4), no SNPs were returned from *CDH23*. This is likely because only a small number of *CDH23* SNPs have been genotyped in ALSPAC. In comparison to *CTIF*, the other candidate genes examined from the syntenic region of human chromosome 18, *ACAA2*, *C18orf32*, *DYM*, *MYO5B* and *SMAD7* (Table 1), did not show effects on hearing, although this could be explained by limitations in the study. The causes of sensorineural hearing loss are not known for individual children in ALSPAC; indeed, this is expected given that ALSPAC is a population study. To date, two genes known to be associated with hearing loss have been specifically examined within the cohort. The most common genetic cause of sensorineural hearing loss is the 35delG mutation of *GJB2*, which encodes the gap junction protein, connexin 26. No children with genotypic data within the cohort are homozygous for 35delG [16]. Also, none of the children in the cohort with the known mitochondrial DNA mutation 1555A- > G have hearing loss [45].

The major strengths of this study are the large number of children with accurate audiometry tests and full genotypic data, resulting in precise associations between SNPs and phenotype. To our knowledge, this is one of few studies to assess genetic contributions to hearing variation in children.

Several limitations are recognised. Due to the genotyping chip used, some of the selected genes were poorly represented by SNPs in ALSPAC to draw information from (e.g., *FSCN-2*, 1 SNP; *HCN2*, 3 SNPs; *ESPNL*, 5 SNPs; *CDH23*, 7 SNPs). Due to the above-mentioned scarcity of genome-wide association studies that have examined hearing in children, replication of our results could not be attempted in a dataset that tested children. The G-EAR dataset used for replication comprised adults with varying degrees of hearing loss, likely to be caused by age and environmental effects. The ALSPAC cohort at age 11 mostly had hearing thresholds within the normal range. The effect sizes of the G-EAR dataset could not be compared to ALSPAC due to differences in analysis methods. It is possible that effects on hearing of other genes studied were not detected due to: small effect sizes; the effect appearing after 11 years of age; the demographics of our study; or that functionally important regions of the genes were not covered by the SNPs examined.

## Conclusions

This study demonstrates, for the first time, suggestive associations of SNPs in *CTIF* and *LCP1* with effects on hearing function in children at age 11. Although these findings were not validated against an independent adult population, there was evidence of a sex-specific locus in *LCP1* being correlated with enhanced low frequency hearing function in girls and adult women. Further studies of *CTIF* and *LCP1* in relation to hearing function and hair cell physiology would be warranted.

## Additional file

Additional file 1:
**Results for all the SNPs included in the analyses of data from ALSPAC or G-EAR.**

## Abbreviations

ALSPAC: Avon Longitudinal Study of Parents and Children
OAE: Otoacoustic emissions
PTA: Pure tone average
SNP: Single nucleotide polymorphism
